# Thyroid Hemiagenesis Associated with Hyperthyroidism

**DOI:** 10.1155/2015/829712

**Published:** 2015-06-22

**Authors:** Gunay Gurleyik, Emin Gurleyik

**Affiliations:** ^1^Department of Surgery, Haydarpasa Numune Education and Research Hospital, 34668 Istanbul, Turkey; ^2^Department of Surgery, Düzce University Faculty of Medicine, 81650 Düzce, Turkey

## Abstract

Thyroid hemiagenesis (TH), very rare congenital anomaly, is generally asymptomatic. We report two cases of TH with hyperthyroidism. *Case One*. The patient presented with signs and symptoms of thyrotoxicosis. Physical examination revealed asymmetric nodular goitre at right lobe. Biochemical analysis revealed the diagnosis of hyperthyroidism. Ultrasound showed multinodular hypertrophy in the right lobe and absence of the left lobe. Nuclear scan, confirming absence of the left lobe, showed hot nodules in the right one. The diagnosis was toxic multinodular goitre. *Case Two*. The thyroid was not palpable in this patient presented with signs and symptoms of thyrotoxicosis. Biochemical analysis revealed the diagnosis of autoimmune thyrotoxicosis. Ultrasound showed mild diffuse hyperplasia of the right lobe and agenesis of the left lobe. Nuclear scan, confirming absence of the left lobe, showed increasing diffuse uptake of radiotracer in the right one. The diagnosis was Graves' disease in this patient. After antithyroid medication, the patients were surgically treated with total excision of the thyroid tissue. TH is sometimes associated with disorders of the thyroid. Hyperthyroidism makes TH cases symptomatic. During evaluation of patients, ultrasound and nuclear scan usually report agenesis of one lobe and establish the diagnosis of TH. The surgical treatment is total removal of hyperactive tissue and total excision of the remaining lobe.

## 1. Introduction

Thyroid hemiagenesis (TH) characterized with total absence of one lobe is a very rare congenital anomaly of the thyroid gland. Patients with TH who have normal thyroid function are usually asymptomatic [1–3]. Therefore, diagnosing of TH in normal population is only possible by screening program using various imaging modalities. On the other hand, TH is usually established during evaluation of patients with thyroid pathology. Functional disorders of the thyroid gland such as hyperactivity make the patient symptomatic.

This report presents two cases of TH discovered during evaluation of two patients with signs and symptoms of hyperthyroidism. Both cases were from an endemic goitre region due to alimentary low iodine intake. Iodine supplementation with iodinated salt and water is used in order to prevent hypothyroidism and goitre formation.

## 2. Report of Two Cases

### 2.1. Case 1

A 49-year-old female patient presented to our clinic with signs and symptoms of hyperthyroidism. Asymmetric hypertrophy of the thyroid gland at right side is determined by inspection. A multinodular goitre is palpated at right side by physical examination.

*Biochemical Analysis*. Blood chemistry demonstrated hyperthyroidism with suppressed thyroid stimulant hormone (TSH = 0.03 uIU/mL), elevated free thyroxin (FT4 = 2.78 ng/dL), and free triiodothyronine (FT3 = 6.85 pg/mL) levels.

*Ultrasonography*. The right lobe (30 × 29 × 57 mm in size) has heterogeneous parenchyma and two solid (35 × 21 and 22 × 16 mm) nodules (multinodular goitre). The left lobe is not visualised (agenesis).

**Figure 1:**
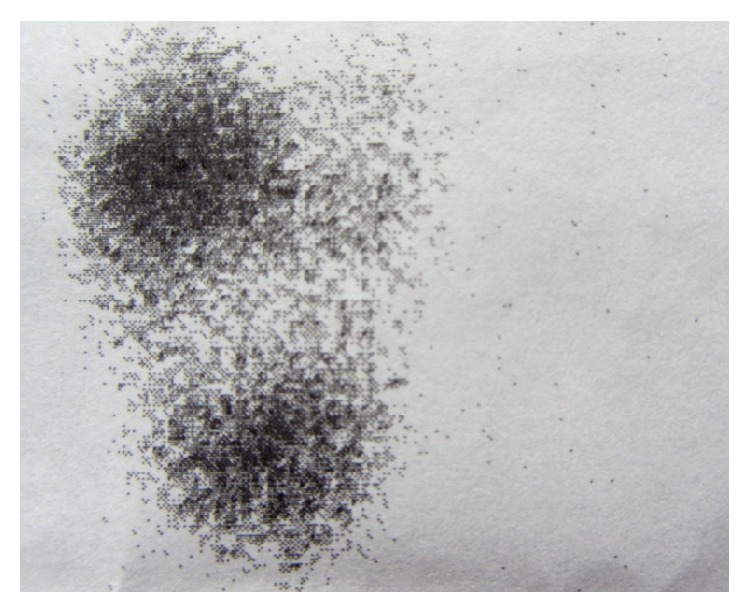
Nuclear scan of case 1. Hypertrophied right lobe and isthmus and absence of the left lobe. Hot nodules are imaged at upper and lower poles of the right lobe.

*Thyroid Nuclear Scan with Tc 99m Pertechnetate*. The left lobes are not visualised (agenesis). Multinodular hypertrophy is imagined in the right lobe of female patient. Nuclear scan identifies two larger hot nodules at upper and lower poles of the right lobe (Figure 1).

*The Pathogenesis*. The case is a middle-aged patient with multinodular goitre due to endemic aetiology. After nodules formation in the thyroid, autonomous hyperactivity of nodules finally resulted in clinical hyperthyroidism. Hemiagenesis was an incidental finding which was discovered by imaging methods during evaluation of the patient who became symptomatic by nodular hyperactivity of the gland. The diagnosis was toxic multinodular goitre in a patient with TH.

The patient received preoperatively antithyroid medical treatment with thyromazol. Antithyroid drug was used until the operation under control of thyroid function tests.

*Surgery*. Total excision of significantly enlarged multinodular right lobe and isthmus was performed in the patient.

*Histopathology*. The size of the right lobe is 60 × 40 × 35 mm which contains two solid nodules (35 × 25 × 25 mm and 5 mm as diameter). The size of isthmus is 30 × 25 × 15 mm which contains one solid nodule of 25 × 15 × 15 mm. The diagnosis is follicular nodular disease.

### 2.2. Case 2

A 25-year-old male patient presented to our clinic with signs and symptoms of hyperthyroidism. The thyroid gland was not palpable.

*Biochemical Analysis*. An autoimmune hyperthyroidism is diagnosed in this case with suppressed TSH = 0.006 uIU/mL and elevated FT4 = 3.45 ng/dL and FT3 = 11.2 pg/mL, antithyroid peroxidase antibody (anti-TPO Ab = 477 IU/mL), and thyrotropin receptor antibody (TR Ab = 46.3 IU/L) levels.

*Ultrasonography*. The right lobe (23 × 27 × 48 mm in size) has heterogeneous parenchyma with some hypo-echoic areas in forms of patches (diffuse hyperplasia). The left lobe of the gland is not visualised (agenesis).

**Figure 2:**
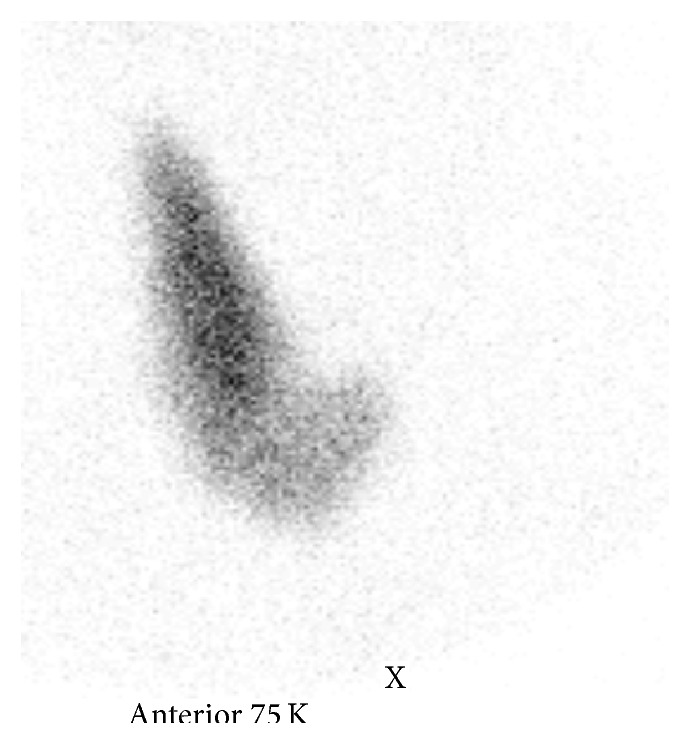
Nuclear scan of case 2. Increased nuclear uptake in the right lobe and absence of the left lobe. The right lobe and the isthmus have a “hockey stick” appearance.

*Thyroid Nuclear Scan with Tc 99m Pertechnetate*. The right lobe and the isthmus are hyperplasic in normal localization. Nuclear activity uptake of the gland is diffusely increased. Diffuse hyperplasic thyroid parenchyma may be related with Graves' disease (Figure 2).

*The Pathogenesis*. This is a young patient who became symptomatic secondary to an autoimmune basis hyperactivity independent of endemic feature of the region. Hemiagenesis was also an incidental finding by imaging methods during evaluation of this patient. The diagnosis was autoimmune toxic diffuse goitre (Graves' disease) in a patient with TH.

The patient received preoperatively antithyroid medical treatment with propylthiouracil and propranolol. Antithyroid drugs were used until the operation under control of thyroid function tests.

*Surgery*. We determined slightly enlarged homogenous right lobe in the patient. The right lobe and the isthmus are totally excised.

*Histopathology*. A thyroid tissue is weighing 27 g after fixation. The size of the right lobe is 90 × 35 × 15 mm. The inner surface is homogenous and rich of colloid. The diagnosis is diffuse hyperplasia.

Our patients are “unilobate” hemiagenesis cases; therefore, unilateral exploration was performed and the remaining “only” thyroidal tissues are totally excised. Superior and inferior parathyroid glands and also recurrent laryngeal nerves were identified at usual anatomical position at the right side in both patients. They were fully exposed and preserved during thyroid surgery. Postoperative period is uneventful. Both patients are discharged at second postoperative day. They are euthyroid with LT4 (100 *μ*g/day) replacement.

## 3. Discussion

Embryological development of the thyroid begins from the endoderm in the primitive pharynx. The thyroid rudiment migrates to usual anatomical position anterior to the thyroid cartilage and the trachea. This rudiment grows laterally to create two lateral lobes of the gland. Hemiagenesis is an incomplete genesis of a lobe that the aetiology remains unclear.

Absence of one lobe, hemiagenesis, is a rare anatomic abnormality of the thyroid gland. The prevalence has been reported between 0.025% and 0.05% in normal population and between 0.16% and 0.25% in patients with thyroid disorders [1–3]. The left lobe is absent in the majority of TH cases; the absence of left lobe has been reported between 70% and 87.5% in such cases [1, 4, 5]. Our patients are two examples of left lobe absence associated with symptomatic disorders of the remaining right lobes. Woman/man ratio is 4–7/1 [1, 5]. One of our patients is an example of rare male cases of TH associated with Graves' disease.

The ultrasound is imaging modality of choice to assess structural feature of the gland. In our patients, agenesis of one lobe has been established first with ultrasound. It has also shown structural changes in the remaining right lobes. The nuclear scan is the modality that establishes functional anatomy of the thyroid. In our cases, functional absence of the left lobe by nuclear scan confirmed its anatomical absence by ultrasound. Nuclear scan has also established hot nodules (multinodular hyperactivity) and increased diffuse uptake (diffuse hyperplasic hyperactivity) in our patients. These two imaging modalities are complementary tools in order to assess structural and functional features of the thyroid and to establish any anatomic abnormality like TH in our patients. Many previous reports have also shown that ultrasound is the first tool for evaluation of thyroid anatomy [1, 2, 6–9]. Many studies have also emphasized importance of nuclear scan in order to assess functional status and to establish functional abnormality of the gland [1, 6, 7, 9, 10]. Some authors have used computer tomography as an imaging modality [6, 8, 11]. In thyroid scintigraphy of our male patient, the right lobe and isthmus of the gland have given “hockey stick” appearance which is pathognomonic image in patients with one lobe and isthmus. Thyroid hemiagenesis with an isthmus present has unique appearance which has been mentioned as “hockey stick sign” [4].

TH has not specific symptoms and signs leading to diagnosis of this abnormality. The remaining lobe of the gland has generally normal function. Usually patients with TH are biochemically euthyroid and clinically asymptomatic [5]. Several thyroid diseases are associated with TH, benign or malignant, and hyper-, normal-, or hypofunctioning disorders [1, 5, 11–16]. This anatomical anomaly is generally established during clinical work-up of symptomatic patients with thyroid disorders.

In a series of TH cases associated with thyroid diseases, hyperthyroidism constitutes only 10% of concomitant disorders of “monolobe (unilobate)” gland [1, 2, 5]. Autonomous hyperactivity of the gland has clinical significance after symptoms of hyperthyroidism. Therefore, toxic goitre has been reported as the reason for complaints of some patients with TH. The majority of other associated diseases with normal thyroid function remain asymptomatic during a long period. Complaints secondary to hyperthyroidism are main reason for evaluation of our patients that biochemical analyses establish the diagnosis of hyperactivity. TH is additional finding during evaluation of our patients by ultrasound and nuclear scan. Hyperthyroidism is one of the functional disorders of the thyroid gland which is surgically managed in the majority of such cases. Proper surgical treatment of hyperthyroidism is total excision of hyperactive thyroidal tissue. Therefore, total thyroidectomy is procedure of choice in patients with toxic multinodular goitre and with Graves' disease. Our patients were “unilobate” hemiagenesis cases that all hyperactive tissues were located in the remaining lobe. In conclusion, unilateral exploration and total excision of the remaining tissues achieved definitive treatment of thyrotoxicosis.

## 4. Conclusions

TH is a rare abnormality which is usually asymptomatic if is not associated with thyroid disorders. Associated hyperactivity of the gland (nodular or diffuse) makes the patient symptomatic. Evaluation of symptomatic patients by ultrasound and nuclear scan establishes TH as an additional finding. Total excision of the remaining lobe provides appropriate treatment of hyperthyroidism. Our patients are rare cases of association of an anatomic abnormality and thyrotoxicosis.
